# Laparoscopic versus conventional appendectomy - a meta-analysis of randomized controlled trials

**DOI:** 10.1186/1471-230X-10-129

**Published:** 2010-11-03

**Authors:** Xiaohang Li, Jialin Zhang, Lixuan Sang, Wenliang Zhang, Zhiqiang Chu, Xin Li, Yongfeng Liu

**Affiliations:** 1 Department of General Surgery, First Affiliated Hospital, China Medical University, Shenyang 110001, Liaoning Province, China; 2 Department of Gastroenterology, First Affiliated Hospital, China Medical University, Shenyang 110001, Liaoning Province, China

## Abstract

**Background:**

Although laparoscopic surgery has been available for a long time and laparoscopic cholecystectomy has been performed universally, it is still not clear whether open appendectomy (OA) or laparoscopic appendectomy (LA) is the most appropriate surgical approach to acute appendicitis. The purpose of this work is to compare the therapeutic effects and safety of laparoscopic and conventional "open" appendectomy by means of a meta-analysis.

**Methods:**

A meta-analysis was performed of all randomized controlled trials published in English that compared LA and OA in adults and children between 1990 and 2009. Calculations were made of the effect sizes of: operating time, postoperative length of hospital stay, postoperative pain, return to normal activity, resumption of diet, complications rates, and conversion to open surgery. The effect sizes were then pooled by a fixed or random-effects model.

**Results:**

Forty-four randomized controlled trials with 5292 patients were included in the meta-analysis. Operating time was 12.35 min longer for LA (95% CI: 7.99 to 16.72, p < 0.00001). Hospital stay after LA was 0.60 days shorter (95% CI: -0.85 to -0.36, p < 0.00001). Patients returned to their normal activity 4.52 days earlier after LA (95% CI: -5.95 to -3.10, p < 0.00001), and resumed their diet 0.34 days earlier(95% CI: -0.46 to -0.21, p < 0.00001). Pain after LA on the first postoperative day was significantly less (p = 0.008). The overall conversion rate from LA to OA was 9.51%. With regard to the rate of complications, wound infection after LA was definitely reduced (OR = 0.45, 95% CI: 0.34 to 0.59, p < 0.00001), while postoperative ileus was not significantly reduced(OR = 0.91, 95% CI: 0.57 to 1.47, p = 0.71). However, intra-abdominal abscess (IAA), intraoperative bleeding and urinary tract infection (UIT) after LA, occurred slightly more frequently(OR = 1.56, 95% CI: 1.01 to 2.43, p = 0.05; OR = 1.56, 95% CI: 0.54 to 4.48, p = 0.41; OR = 1.76, 95% CI: 0.58 to 5.29, p = 0.32).

**Conclusion:**

LA provides considerable benefits over OA, including a shorter length of hospital stay, less postoperative pain, earlier postoperative recovery, and a lower complication rate. Furthermore, over the study period it was obvious that there had been a trend toward fewer differences in operating time for the two procedures. Although LA was associated with a slight increase in the incidence of IAA, intraoperative bleeding and UIT, it is a safe procedure. It may be that the widespread use of LA is due to its better therapeutic effect.

## Background

Acute appendicitis is a common indication for abdominal surgery with a life-time incidence between 7 and 9% [1], and appendectomy is one of the most common surgical procedures. Open appendectomy (OA) performed through the right lower quadrant incision was first described in 1894 [2]. It has become the standard treatment of choice for acute appendicitis, remaining mainly unchanged for 100 years due to its favorable efficacy and safety. Laparoscopic appendectomy (LA), first performed by Semm [3] in 1983, has gradually gained acceptance. However, there remains a continuing controversy in the literature regarding the most appropriate method of removing the inflamed appendix.

Considering research published in English, to date there have been some prospective randomized controlled studies comparing LA and OA. While some studies concluded that LA was superior to OA in terms of a faster recovery, improved wound healing, and earlier resumption of diet, other studies found no such benefits, or even favored conventional appendectomy. However, most of these studies had small sample sizes, and therefore the risk of a type II error (failing to observe a difference when in truth there is one) may be high. The statistical power of analysis can be increased through a meta-analysis, which combines and compares the data from different studies. The most recent meta-analysis of the two techniques was published 3 years ago [4], and many high-quality trials have been published since then. Therefore, we performed a new meta-analysis to determine which technique, LA or OA, gives better patient outcome.

## Methods

### Study search

We searched electronic databases (MEDLINE, EMBASE, and the Cochrane Central Register of Controlled Trials) for potentially relevant randomized controlled trials comparing LA and OA conducted from January 1990 to December 2009, published in English. Keywords used for the search included: randomized controlled trial and appendectomy/appendicectomy, laparoscopic appendectomy/appendicectomy, laparoscopic versus open appendectomy/appendicectomy, minimally invasive versus conventional appendicectomy/appendectomy, and laparoscopic and open appendectomy/appendicectomy. The "related articles" function was used to broaden the search, and reference lists of these studies were reviewed to determine whether any other trials were potentially eligible for inclusion in the meta-analysis. Additionally, the abstracts from main international meetings (including the Society of American Gastrointestinal Endoscopic Surgeons, the European Association for Endoscopic Surgery and the International Pediatric Endosurgery Group) for the last 5 years were searched by manual retrieval, and the authors were asked to provide full information on their study using a detailed data extraction form. Two investigators independently reviewed all studies. Eligible trials were then selected according to the inclusion criteria below. Discrepancies were resolved, if necessary, by discussion and consulting a senior reviewer.

### Assessment of study eligibility

We systematically reviewed each study according to the following criteria: (1) a prospective randomized study format only; (2) a comparison of laparoscopic and open appendicectomy; (3) The study reported at least one of the desirable outcomes mentioned below and the standard deviation of the mean for continuous outcomes of interest was reported or can be calculated; (4) Studies that allocated patients depending on the availability of staff or instruments were excluded; (5) Studies that used variations on the standard laparoscopic technique, including hybrid procedures or single trochar techniques, were also excluded.

### Outcomes of interest

The outcomes of interest included operating time, postoperative length of hospital stay, resumption time of normal activities and diet, and postoperative pain (assessed by visual analogue scale (VAS) graded from 0 to 10, with 0 being no pain and 10 being the most intense pain). We were also interested in several complications including: wound infection, postoperative ileus, intraoperative bleeding (>500 mL), urinary tract infection (UTI) and intra-abdominal abscess (IAA) formation following LA vs. OA techniques.

### Data extraction and quality assessment

Two investigators independently extracted the following data from each included study: first author, country, year of publication, number of participants allocated to each intervention group, study population characteristics, severity of appendicitis, the desirable outcomes, the method of randomization and allocation concealment, the blinding of outcome assessment, conversion rate from LA to OA, and whether intention-to-treat analysis was used. Any disagreement was discussed and resolved by consensus. The Jadad scale [5] was used to evaluate the overall quality of all included articles. According to Kjaergard et al.'s recommendation [6], low-quality studies have a score of ≤ 2 and high-quality studies have a score of ≥ 3.

### Statistical analysis

Meta-analysis was performed using Cochrane Collaboration Review Manager 5.0 software. For continuous variables, statistical analysis was carried out using the weighted mean difference (WMD) as the summary statistic, comparing the treatment (LA) group with the reference (OA) group using the inverse variance method. A negative WMD favored the LA group, and the point estimate of the WMD was considered statistically significant at the p < 0.05 level if the 95% confidence interval (CI) did not include the value zero. For categorical variables, statistical analysis was carried out using the odds ratio (OR) as the summary statistic. An OR of less than one favored the LA group, and the point estimate of the OR was considered statistically significant at the p < 0.05 level if the 95% CI did not include the value one.

Fixed effects models were initially calculated for all outcomes. We then tested for homogeneity among the studies by calculating the I^2 ^which describes the proportion of total variation in study estimates that is due to heterogeneity. If the test rejects the assumption of homogeneity of studies, then it is not appropriate to use a fixed effects model [7], and random effects analysis should be performed. Sensitivity analyses were conducted to explore statistical heterogeneity. Whether there was publication bias from a fixed effects model could be evaluated by an inverted funnel plot.

If studies reported their continuous variables as medians with ranges that a meta-analysis can not use, we assumed that the mean is equal to the median value itself and estimated the standard deviation (SD) as a quarter of the range (samples ≤ 70) or range/6 (samples > 70) [8]. If neither ranges nor any other measure of dispersion was reported, and it was impossible to estimate the mean and SD based on the published data, the corresponding continuous variables were excluded from the statistical pool.

To ensure finding the exact change in comparative outcomes between OA and LA, and because the LA procedure is relatively recent and experience with it has increased rapidly, the studies published before the year 2000 were analyzed separately from those published afterward.

## Results

### Search results

**Figure 1:**
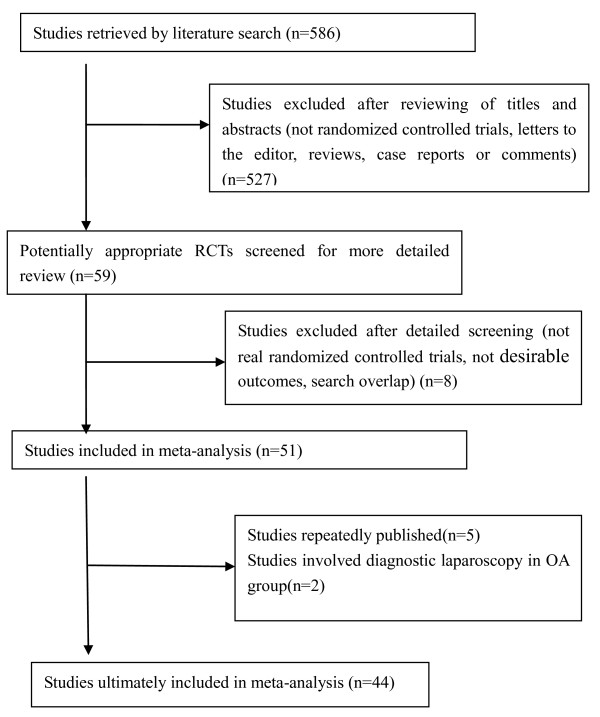
**Identification of studies for inclusion**.

The search strategy generated 586 studies. After the initial screening, 51 studies were thought to meet the inclusion criteria [9-59]. After further screening of full texts, it was found that in three instances three [17,55,56], two [24,59] and three [31,57,58] of the studies were performed by the same author; we therefore chose the most recent or highest quality article [17,24,31] from each author. Two studies which involved the use of diagnostic laparoscopy followed by OA in the LA group were also excluded [53,54]. In the end, 44 randomized controlled trials [9-52] were identified for consideration in the meta-analysis (Figure 1). A total of 5292 participants were enrolled in the 44 studies, of which 2609 (49.30%) underwent LA and 2683 (50.70%) OA. The characteristics of these studies are listed in Additional file 1, Table S1.

### Methodological quality assessment of studies included in our meta-analysis

Additional file 1, Table S2 shows the quality of the included studies as assessed by the Jadad scale.

### Effects of the intervention

#### Operating time

Thirty-six studies reported data that allowed quantitative pooled analysis for operating time (Additional file 1, Table S3). According to the analysis of all 36 with a random effects model, the laparoscopic approach takes 12.35 min longer than open surgery (95% CI: 7.99 to 16.72, p < 0.00001). However, subgroup analysis revealed that pre-2000, LA took 15.14 min longer than OA (95% CI: 10.79 to 19.50, p < 0.00001), and this decreased to only 8.67 minutes after this period (95% CI: 0.48 to 16.86, p = 0.04).

#### Postoperative hospital stay

Thirty-two studies comparing LA vs. OA reported postoperative length of stay. Using a random effects model, the pooled data from all years showed that the laparoscopic approach led to a reduction in postoperative stay of 0.60 days (95% CI: -0.85 to -0.36, p < 0.00001; Additional file 1, Table S3). The clinical significance of this was not clear. During the pre-2000 period, the difference in postoperative stay was only 0.48 days (95% CI: -0.77 to -0.19, p = 0.001), and increased to 0.75 days from the year 2000 onwards (95% CI: -1.10 to -0.39, p < 0.0001). The evidence of a nearly one day hospital stay reduction would appear to have a stronger clinical significance.

#### Return to normal activity

Twenty-one studies reported the time required for patients to return to normal activity. Using a random effects model, the results showed an overall 4.52-day reduction in recovery time for LA compared to OA (95% CI: -5.95 to -3.10, p < 0.00001; Additional file 1, Table S3). For the subgroup of studies reported during the year 2000 and after, there was no statistical difference between the two procedures, although the results demonstrated a trend in favor of LA.

#### Resumption of normal diet

Thirteen studies reported the time until patients could tolerate a normal diet. Overall analysis with a random effects model showed that the laparoscopic approach led to a reduction in this period of 0.34 days as compared to OA (95% CI: -0.46 to -0.21, p < 0.00001; Additional file 1, Table S3). The clinical significance of this was not clear. Subgroup analysis demonstrated a trend in favor of LA which had increased over time.

#### Postoperative pain

Only eight studies recorded postoperative pain data, measured by VAS, on the first postoperative day. Meta-analysis of VAS for postoperative pain with a random effects model demonstrated a score of 0.70 points less for LA compared with OA (95% CI: -1.22 to -0.19, p = 0.008; Additional file 1, Table S3). Subgroup analysis demonstrated a trend in which the VAS difference between LA and OA became less, decreasing from -1.11 for pre-2000 studies (95% CI: -1.80 to -0.42, p = 0.002) to -0.11 for post-2000 studies (95% CI: -1.05 to 0.83, p = 0.82).

#### Wound infection

Thirty-one studies reported the incidence of postoperative wound infection. Meta-analysis with a fixed effects model showed a 3.81% (76/1994) incidence of wound infection for LA, compared with 8.41% (174/2069) for OA. The difference was statistically significant (OR = 0.45, 95% CI: 0.34 to 0.59, p < 0.00001; Additional file 1, Table S4). Analyzing the pre- and post-subgroups separately, this statistical significance continued, whereby the risk of wound infection associated with the LA procedure was demonstrably less than for the OA (OR = 0.36, 95% CI: 0.23 to 0.54, p < 0.00001 and OR = 0.53, 95% CI: 0.37 to 0.76, p = 0.0005, respectively).

#### IAA

Seventeen studies recorded data for the incidence of IAA. Pre-2000, post-2000, and overall meta-analysis with a fixed effects model failed to show any statistically significant difference in the incidence of IAA, but there was a trend in favor of OA (Additional file 1, Table S4).

#### Postoperative ileus

The combined data from 18 studies showed that the incidence of postoperative ileus was 1.96% (32/1630) for OA, and 1.78% (28/1576) for LA. The effect size of the difference in the ORs, analyzed by a fixed effects model, was 0.91 (95% CI: 0.57 to 1.47, p = 0.71; Additional file 1, Table S4). Although the results indicated that the laparoscopic approach resulted in a reduced incidence of postoperative ileus, the difference was not statistically significant.

#### Intraoperative bleeding

Only four studies provided details of intraoperative bleeding (>500 mL). The meta-analysis with a fixed effects model suggested that the conventional approach led to a reduced incidence of intraoperative bleeding, however, the difference was not statistically significant (OR = 1.56, 95% CI: 0.54 to 4.48, p = 0.41; Additional file 1, Table S4). Because there was only one pre-2000 study, we did not perform the subgroup analysis.

#### Urinary tract infection

The combined data from only five studies revealed that the incidence of postoperative UTI was 0.76% (3/395) for OA and 1.75% (7/401) for LA. The effect size of the difference in ORs, analyzed by the fixed effects model, was 1.76 (95% CI: 0.58 to 5.29, p = 0.32; Additional file 1, Table S4). Although the results indicated that the laparoscopic approach resulted in an incidence of postoperative UTI that was greater than that of OA, the difference was not statistically significant.

#### Sensitivity analysis

The heterogeneity was shown significantly for operating time, hospital stay, return to full activity, resumption of normal diet, and VAS for postoperative pain. A sensitivity analysis was performed by excluding studies that intention-to-treat analysis (ITT) was not adopted or unclear, or included only males or females or children, or reported no measure of the standard deviation. Then heterogeneity was decreased. As shown in Additional file 1, Table S3, the overall estimates were similar between the sensitivity analysis and the meta-analysis.

#### Publication bias assessment

**Figure 2:**
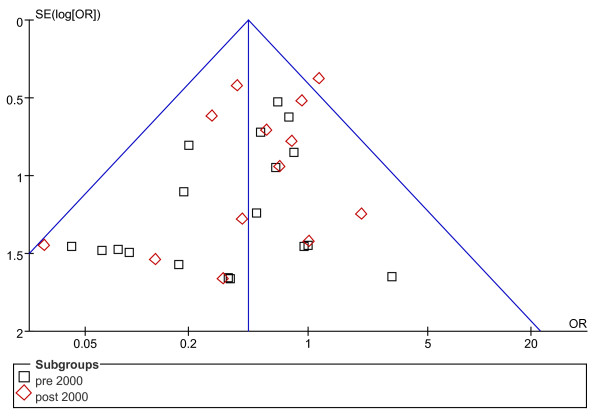
**Inverted funnel plot analysis of the wound infection rate between LA and OA**.

**Figure 3:**
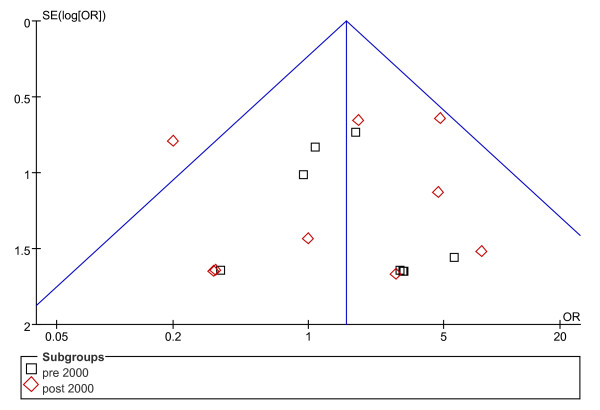
**Inverted funnel plot analysis of the intra-abdominal abscess rate between LA and OA**.

**Figure 4:**
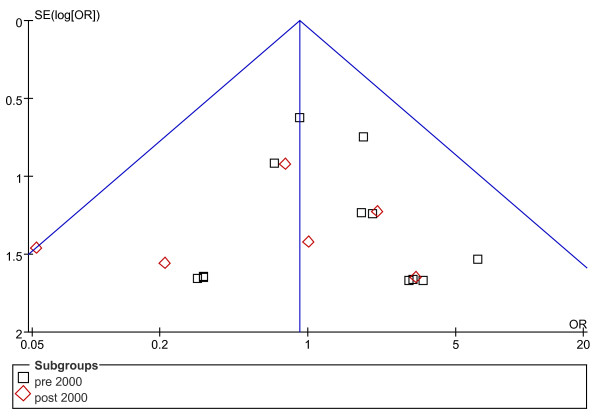
**Inverted funnel plot analysis of the postoperative ileus rate between LA and OA**.

Concerning rates of complications, the possibility of publication bias was analyzed by the inverted funnel plot. The plot resembles a symmetric inverted funnel (the 95% CI). It is notable that in Figure 2 and 3, illustrating the inverted funnel plot analyses of wound infection and IAA rates respectively, only one study lay outside the 95% CI axis. In Figure 4, the inverted funnel plot analysis of the postoperative ileus rate, there was no study outside the 95% CI axis. We therefore conclude that there is no evidence of publication bias in our analysis.

## Discussion

Laparoscopy, as a minimally invasive technique, has unique advantages in several areas, and many scholars have tried to prove these advantages. Yet, because OA involves a small incision and perfect skill, the advantages of LA over OA continues to be debated. In order to confirm the greater efficacy of LA, we performed the present research. Compared to other meta-analyses [60-62], our's included more high-quality studies and analyzed more outcomes. Therefore, the therapeutic effects of LA and OA can be sufficiently evaluated here.

Regarding operating time, there was an obvious trend toward parity between the two procedures. A reputation for extended operating time is a major disadvantage, and has considerably influenced the widespread use of LA: according to an initial study, LA involved a significant increase in operating time [45]. This may have been due to the inexperience of the surgeons with the new technique. However, with increased experience the mean operating time for LA and OA become similar [63].

According to the analysis for the entire study period (1990-2009), the length of hospital stay after surgery was shortened in LA by 0.60 days, a difference that is not of clinical significance. However, since the year 2000 the reduction in postoperative hospital stay became more significant. A 48-hour discharge policy for LA proposed by Grewal et al. [64] contributed to the increased difference. However, our results are not consistent with that of others [24], who believe that this was one area where LA has no advantage over OA. The discrepancy may be due to the different social standards, insurance systems and health care policies.

Early return to full activity is accepted as an obvious advantage of LA, which was supported by a large scale meta-analysis conducted by the Cochrane Colorectal Cancer Group [65]. The trocar incisions of LA contribute to minimal trauma to the abdominal wall and less pain [66], allowing faster recovery. A trend towards less difference in return to normal activity was noted in our study (OR = -5.73 for pre-2000 studies vs. -2.32 for post-2000 studies). Lord and Sloane [67] considered that early discharge and mobilization after OA were also feasible, if given the appropriate infrastructure. Fast resumption of a normal diet following LA was another appealing advantage, resulting from minimal manipulation of the cecum and ileum [68]. Although a significant difference was found (p < 0.00001), the practicality of a difference of 0.34 days remains doubtful.

Postoperative pain can be assessed quantitatively by the daily requirements for analgesics. Nevertheless, the various kinds of analgesics and routes of administration make it difficult to estimate pain relief. We qualitatively assessed pain on the first postoperative day by means of a VAS. The meta-analysis indicated that LA offered significant advantages in relieving postoperative pain (p = 0.008), mainly due to its minimal invasiveness. However, the difference in VAS between the two procedures was not statistically significant for the post-2000 subgroup of studies (p = 0.82). The trend toward a smaller incision in OA may explain this decreased difference.

The reduction of wound infection is a significant advantage of LA. The chance of wound infection is greater in OA partly because the inflamed appendix is removed from the abdominal cavity directly through the wound, whereas in LA it is extracted via a bag or trocar. In addition, the port-site wounds in LA are smaller compared to the longer wounds of OA, especially in obese patients.

There were several explanations for the reduction of ileus following LA. Firstly, decreased handling of the bowel during the procedure leads to less postoperative adhesion, and such adhesion may be responsible for ileus. Secondly, patients after LA had less opiate analgesics, which inhibited bowel movements in the postoperative period. Lastly, earlier mobilization after LA may also contribute to the reduction of adhesion.

The finding that the incidence of IAA, intraoperative bleeding and UTI after LA was higher compared with OA should be noteworthy, even though these were not statistically significant. Intraoperative bleeding and UTI were rare complications after appendectomy. The reason why the rates were higher after LA is not clear, and the further investigation is necessary. IAA is a serious complication following appendectomy and can potentially be life threatening; many investigators pay close attention to this complication. Gupta et al. [69] considered that aggressive manipulation of the infected appendix and increased use of irrigation fluid, possibly producing greater contamination of the peritoneal cavity, might have an impact on IAA formation after LA. Memon et al. [70] believed that carbon dioxide pneumoperitoneum contributed to the mechanical diffusion of bacteria inside the peritoneal cavity, but experimental proof of this is lacking. Brümmer et al. [71] thought that the risk of developing IAA after LA was greater than for OA (0.31% vs. 0.21%). Conversely, Katkhouda et al. [72] believed that mastery of the learning curve and the use of standardized surgical techniques reduced the incidence of IAA after LA.

It is hard to understand why the conversion rate from LA to OA increased over time. Perhaps as experience with the laparoscopic procedure is gained, surgeons might attempt to perform LA for complicated cases such as gangrenous and perforated appendicitis, most of which might have been treated previously by the open approach. The increased difficulty in LA might have resulted in the higher conversion rate.

Meta-analysis is an increasingly popular method of data analysis to solve debatable problems. However, there were some drawbacks in our research. First, different studies included in our research may have had slightly different defining criteria for the outcome measures, e.g., wound infection and ileus. Resumption of normal activity and diet might also be defined differently. Second, not all the studies measured data based on a double-blind. In the absence of a double-blind, subjective variables such as pain assessment could be considerably influenced by the enthusiasm for a novel technique. Third, there was variation in surgical techniques and treatment protocols amongst the studies, and therefore heterogeneity in the studies might exist. Fourth, although most studies were comparable with regard to age and sex, fewer were matched for severity of appendicitis (represented by fever, raised WBC, peritonitis and perforated rate) and weight. These considerations may have an influence on postoperative complications, recovery and operation time.

Despite the limitations mentioned above, we believe that LA involves a shorter hospital stay, less postoperative pain, a faster recovery, and a lower complication rate compared with OA. Our study demonstrates that LA is a safe and effective treatment alternative for patients with acute appendicitis, and is recommended for those hospitals where laparoscopic expertise and equipment are available. Although LA was associated with slightly more operating time than OA, subgroup analysis revealed that this difference has been diminishing. The slightly higher incidence of IAA, intraoperative bleeding and UTI was worrisome, and due to insufficient primary data our research unfortunately was unable to stratify the postoperative abscess rate according to the severity of the appendicitis. Therefore, bias between groups might be present and might have affected the results. Further studies should match for the severity of appendicitis to settle the question.

## Conclusion

In conclusion, the present study shows that LA provides considerable benefits over OA, including a shorter hospital stay, less postoperative pain, earlier postoperative recovery, and lower complication rate. Therefore, the widespread use of LA is routinely recommended in those hospitals where laparoscopic expertise and equipment are available.

## Competing interests

The authors declare that they have no competing interests.

## Authors' contributions

XHL: collected, analyzed, interpreted data, wrote the manuscript. JLZ: planned, designed and corrected the manuscript. LXS: analyzed, interpreted data, and helped to draft the manuscript. WLZ: provided general advice, analyzed and interpreted data. ZQC: provided general advice, analyzed and interpreted data. XL: interpreted data, helped to correct the manuscript. YFL: participated in study design and revising the manuscript. All authors read and approved the final manuscript.

## Pre-publication history

The pre-publication history for this paper can be accessed here:

http://www.biomedcentral.com/1471-230X/10/129/prepub

## Supplementary Material

Additional file 1 **4 tables**. This additional file includes 4 large tables with the format of Microsoft Word. The titles of dataset are listed below: Table S1: Characteristics of 44 studies included in our meta-analysis. Table S2: Methodological quality of studies included in our meta-analysis. Table S3: Meta-analysis of different outcomes in all studies and in selective studies. Table S4: Meta-analysis of the complications in all studies Click here for file
