# Supplementary material for: Laparoscopic versus conventional appendectomy - a meta-analysis of randomized controlled trials
Source: BMC Gastroenterol. 2010 Nov 3;10:129. doi: 10.1186/1471-230X-10-129 (PMC2988072; doi:10.1186/1471-230X-10-129)
Supplement: Additional file 1 — 4 tables. This additional file includes 4 large tables with the format of Microsoft Word. The titles of dataset are listed below: Table S1: Characteristics of 44 studies included in our meta-analysis. Table S2: Methodological quality of studies included in our meta-analysis. Table S3: Meta-analysis of different outcomes in all studies and in selective studies. Table S4: Meta-analysis of the complications in all studies [file 1471-230X-10-129-S1.DOC]

**Table S1: Characteristics of 44 studies included in our meta-analysis**

| Study | Study country | Year | Case | | Mean Age(y) | | Sex(male/female) | | Intention to treat analysis | Conversion  Rate(from LA to OA) | Matching^**^ |
| --- | --- | --- | --- | --- | --- | --- | --- | --- | --- | --- | --- |
|  |  |  | LA | OA | LA | OA | LA | OA |  |  |  |
| Al-Mulhim AS^9^ | Saudi Arabia. | 2002 | 30 | 30 | 23 | 26 | all woman | all woman | ― | 5% | 2,4,5,6,11 |
| Attwood SE^10^ | Ireland | 1992 | 30 | 32 | 21 ^*^ | 27 ^*^ | ― | ― | yes | 6.67% | 5 |
| Bruwer F^11^ | South Africa | 2003 | 18 | 16 | ― | ― | all woman | all woman | yes | 5.56% | 5 |
| Cox MR^12^ | Australian | 1996 | 33 | 31 | 25 ^*^ | 25 ^*^ | all man | all man | yes | 15% | 2,4,5 |
| Frazee RC^13^ | USA | 1994 | 38 | 37 | 30 | 28 | 16/22 | 20/17 | Yes | 5% | 5,7 |
| Hansen JB^14^ | Australian | 1996 | 84 | 74 | 25 ^*^ | 22 ^*^ | 32/52 | 25/49 | no | 8.33% | 5,7,9 |
| Hart R^15^ | Canadian | 1996 | 44 | 37 | 44 | 37 | 29/15 | 21/16 | no | 9% | 5,7 |
| Heikkinen TJ^16^ | Finland | 1998 | 19 | 21 | 34 ^*^ | 37 ^*^ | 9/10 | 12/9 | no | 5% | 1,2,4,5,6,7,8,11 |
| Hellberg A^17^ | Sweden | 1999 | 244 | 256 | ― | ― | ― | ― | yes | 12% | 5,6,7 |
| Helmy MA^18^ | Egypt | 2001 | 50 | 50 | 29 ^*^ | 27 ^*^ | all man | all man | ― | 18% | 2,5 |
| Huang MT^19^ | Taiwan | 2001 | 23 | 26 | 32 | 34 | ― | ― | none | 0% | 4,7 |
| Ignacio RC^20^ | USA | 2004 | 26 | 26 | 29 | 27 | all man | all man | yes | 3.84% | 5 |
| Kald A^21^ | Sweden | 1999 | 49 | 50 | 24 ^*^ | 32 ^*^ | 28/21 | 21/29 | yes | 2.04% | 11 |
| Kaplan M^22^ | Tuikey | 2009 | 50 | 50 | 24 | 26 | 33/17 | 31/19 | none | 0% | 5,7 |
| Karadayi KT^23^ | Tuikey | 2003 | 30 | 30 | 29 | 29 | 14/16 | 20/10 | no | 10% | 5,7 |
| Kathouda N^24^ | USA | 2005 | 113 | 134 | 29 ^*^ | 28 ^*^ | 78/35 | 104/30 | yes | 7.96% | 2,5,7,11 |
| Kazemier G^25^ | Netherlands | 1997 | 97 | 104 | 32 | 32 | 52/45 | 59/45 | yes | 11.54% | 4,5,7,11 |
| Klingler A^26^ | Australian | 1998 | 87 | 82 | 30 ^*^ | 24 ^*^ | 44/43 | 39/43 | none | 0% | 2,5,6,7,11 |
| Kum CK^27^ | Singopore | 1993 | 52 | 57 | 33 | 31 | 15/37 | 19/38 | none | 0% | 5,6,7 |
| Laine S^28^ | Finland | 1997 | 25 | 25 | 27 | 28 | all women | all women | yes | 8% | 5 |
| Lavonius MT^29^ | Finland | 2001 | 23 | 20 | 13 ^*^ | 12 ^*^ | ― | ― | yes | 4.35% | 1,2,4,5 |
| Lejus C^30^ | France | 1996 | 32 | 31 | 11 | 11 | ― | ― | none | 0% | 11 |
| Lintula H^31^ | Finland | 2004 | 43 | 44 | 11 | 12 | 27/16 | 27/17 | none | 0% | 5,6,7,11 |
| Little DC^32^ | USA | 2002 | 44 | 44 | 12 | 11 | ― | ― | yes | 6.81% | 11 |
| Long KH^33^ | USA | 2001 | 93 | 105 | ― | ― | ― | ― | yes | 16% | 1,2,4,5,7,8,11 |
| Macarulla E^34^ | Spain | 1997 | 106 | 104 | 27 | 29 | 42/64 | 48/56 | yes | 8.49% | 5,7,9,10 |
| Martin LC^35^ | USA | 1995 | 81 | 88 | 27 | 29 | 51/37 | 49/32 | yes | 16.05% | 5,7,10 |
| Milewczyk M^36^ | Poland | 2003 | 96 | 104 | 26 | 28 | 52/44 | 59/45 | yes | 8.33% | 2,4,5,7,11 |
| Minne L^37^ | USA | 1997 | 27 | 23 | 32 ^*^ | 31 ^*^ | 18/9 | 13/10 | yes | 7.41% | 5,7,10 |
| Moberg AC^38^ | Sweden | 2005 | 81 | 82 | 31 ^*^ | 31 ^*^ | 46/35 | 58/24 | yes | 2.47% | 5,6,7 |
| Moirangthem GS^39^ | Indian | 2008 | 25 | 25 | 31 | 35 | 6/19 | 17/8 | none | 0% | 5 |
| Mutter D^40^ | France | 1996 | 50 | 50 | 29 | 27 | all man | all man | yes | 12% | 1,2,5 |
| Nordentoft T^41^ | Denmark | 2000 | 12 | 11 | 40 | 33 | ― | ― | no | 8.33 | 5,10 |
| Ortega AE^42^ | USA | 1995 | 78 | 86 | 26 | 25 | 51/26 | 67/19 | Yes | 7.69% | 2,5,6,7,9 |
| Ozmen MM^43^ | Turkey | 1995 | 35 | 35 | 23 | 28 | 17/18 | 15/20 | none | 0% | 4,5,7 |
| Pedersen AG^44^ | Denmark | 2001 | 282 | 301 | 26 ^*^ | 27 ^*^ | 131/151 | 143/158 | yes | 23.04% | 5,6,7 |
| Reiertsen O^45^ | Norway | 1997 | 42 | 42 | 34 | 33 | 31/11 | 26/16 | none | 0% | 4,5,7,11 |
| Ricca R^46^ | USA | 2007 | 28 | 24 | 31 | 31 | ― | ― | no | 3.57% | 5,6,11 |
| Simon P^47^ | Germany | 2009 | 20 | 20 | 12 | 11 | ― | ― | none | 0% | 1,2,4,5,6 |
| Tate JJ^48^ | Hongkong | 1993 | 70 | 70 | 31 | 33 | 43/27 | 43/27 | Yes | 20% | 4,5,6,7,8 |
| Tzovaras G^49^ | Greece | 2007 | 38 | 40 | 26 ^*^ | 22 ^*^ | all man | all man | yes | 18.50% | 5,6 |
| Wei HB^50^ | China | 2009 | 112 | 108 | 29 | 27 | 67/45 | 66/42 | none | 0% | 1,2,5,6,7 |
| Williams MD^51^ | USA | 1996 | 19 | 18 | 28 | 26 | 14/5 | 14/4 | no | 10.53% | 1,2,3,4,5,6,7,9 |
| Yin WY^52^ | Taiwan | 1996 | 30 | 40 | 36 | 36 | 16/14 | 21/19 | yes | 3.33% | 2,4,5,6,7 |

*median

**Matching: 1, fever; 2, white blood cell count(WBC); 3, peritonitis; 4, duration; 5, age; 6, weight; 7, sex; 8, classification of occupation; 9, previous laparotomy; 10, perforated rate; 11, type of appendiceal inﬂammation.

**Table S2:** Methodological quality of studies included in our meta-analysis

| Studies included  in this research | Randomization method | Explanation for  withdrawals/drop-outs | Allocation  concealmen | Blind | Jadad  score |
| --- | --- | --- | --- | --- | --- |
| Al-Mulhim AS^9^ | unclear | yes | appropriate | unclear | 3 |
| Attwood SE^10^ | unclear | yes | appropriate | unclear | 3 |
| Bruwer F^11^ | computer-generated | yes | appropriate | unclear | 4 |
| Cox MR^12^ | unclear | yes | unclear | unclear | 2 |
| Frazee RC^13^ | unclear | yes | unclear | unclear | 2 |
| Hansen JB^14^ | random number tables | yes | appropriate | unclear | 4 |
| Hart R^15^ | computer-generated | yes | appropriate | unclear | 4 |
| Heikkinen TJ^16^ | unclear | yes | appropriate | unclear | 3 |
| Hellberg A^17^ | computer-generated | yes | appropriate | unclear | 4 |
| Helmy MA^18^ | unclear | yes | unclear | unclear | 3 |
| Huang MT^19^ | unclear | yes | unclear | unclear | 2 |
| Ignacio RC^20^ | unclear | yes | unclear | measures taken | 4 |
| Kald A^21^ | computer-generated | yes | appropriate | unclear | 4 |
| Kaplan M^22^ | unclear | yes | unclear | unclear | 2 |
| Karadayi KT^23^ | computer-generated | yes | appropriate | unclear | 4 |
| Kathouda N^24^ | computer-generated | yes | appropriate | measures taken | 5 |
| Kazemier G^25^ | unclear | yes | appropriate | unclear | 3 |
| Klingler A^26^ | unclear | yes | unclear | unclear | 2 |
| Kum CK^27^ | coin toss | yes | unclear | unclear | 3 |
| Laine S^28^ | unclear | yes | unclear | unclear | 2 |
| Lavonius MT^29^ | unclear | yes | appropriate | unclear | 3 |
| Lejus C^30^ | unclear | yes | unclear | measures taken | 4 |
| Lintula H^31^ | unclear | yes | appropriate | measures taken | 4 |
| Little DC^32^ | unclear | yes | appropriate | unclear | 3 |
| Long KH^33^ | random number tables | yes | appropriate | unclear | 4 |
| Macarulla E^34^ | unclear | yes | appropriate | unclear | 3 |
| Martin LC^35^ | unclear | yes | unclear | unclear | 2 |
| Milewczyk M^36^ | random number tables | yes | unclear | unclear | 3 |
| Minne L^37^ | 10 repeating random  numbers on a master list | yes | unclear | unclear | 3 |
| Moberg AC^38^ | computer-generated | yes | appropriate | measures taken | 5 |
| Moirangthem GS^39^ | unclear | yes | unclear | unclear | 2 |
| Mutter D^40^ | unclear | yes | unclear | unclear | 2 |
| Nordentoft T^41^ | unclear | yes | unclear | unclear | 2 |
| Ortega AE^42^ | unclear | yes | unclear | measures taken | 4 |
| Ozmen MM^43^ | unclear | yes | unclear | unclear | 2 |
| Pedersen AG^44^ | computer-generated | yes | appropriate | unclear | 4 |
| Reiertsen O^45^ | unclear | yes | unclear | unclear | 2 |
| Ricca R^46^ | unclear | yes | unclear | measures taken | 4 |
| Simon P^47^ | pulling lots | yes | unclear | unclear | 3 |
| Tate JJ^48^ | computer-generated | yes | appropriate | unclear | 4 |
| Tzovaras G^49^ | unclear | yes | unclear | unclear | 2 |
| Wei HB^50^ | unclear | yes | unclear | unclear | 2 |
| Williams MD^51^ | unclear | yes | unclear | unclear | 2 |
| Yin WY^52^ | random number tables | yes | appropriate | measures taken | 5 |

**Table S3: Meta-analysis of different outcomes in all studies and in selective studies**

| Outcome | Studies reporting outcome | | Meta-analysis | | | | | | | |
| --- | --- | --- | --- | --- | --- | --- | --- | --- | --- | --- |
|  | Studies | Patients | Type | Mean difference | | 95% CI | | P | | I^2^ (%) |
| **All studies**  Operating time | 36 | 4055 | Random | 12.35 | | 7.99,16.72 | | <0.00001 | | 97 |
| Pre 2000 | 21 | 2526 | Random | 15.14 | | 10.79,19.50 | | <0.00001 | | 95 |
| Post 2000 | 12 | 1529 | Random | 8.67 | | 0.48,16.86 | | 0.04 | | 98 |
| Postoperative hospital stay | 32 | 3839 | Random | -0.60 | | -0.85,-0.36 | | <0.00001 | | 96 |
| Pre 2000 | 18 | 2445 | Random | -0.48 | | -0.77,-0.19 | | 0.001 | | 94 |
| Post 2000 | 14 | 1394 | Random | -0.75 | | -1.10,-0.39 | | <0.0001 | | 95 |
| Return to normal activity | 21 | 2383 | Random | -4.52 | | -5.95,-3.10 | | <0.0001 | | 94 |
| Pre 2000 | 14 | 1687 | Random | -5.73 | | -7.48,-3.99 | | <0.0001 | | 93 |
| Post 2000 | 7 | 695 | Random | -2.32 | | -4.89,0.26 | | 0.08 | | 93 |
| Resumption of normal diet | 13 | 1772 | Random | -0.34 | | -0.46,-0.21 | | <0.00001 | | 96 |
| Pre 2000 | 6 | 888 | Random | -0.16 | | -0.29,-0.03 | | 0.02 | | 71 |
| Post 2000 | 7 | 884 | Random | -0.41 | | -0.60,-0.23 | | <0.0001 | | 97 |
| VAS for postoperative pain | 8 | 1504 | Random | -0.70 | | -1.22,-0.19 | | 0.008 | | 95 |
| Pre 2000 | 5 | 1105 | Random | -1.11 | | -1.80,-0.42 | | 0.002 | | 91 |
| Post 2000 | 3 | 399 | Random | -0.11 | | -1.05,0.83 | | 0.82 | | 96 |
| **Studies that adopted ITT**  **or reported the standard deviation** | | | | | | | | | | |
| Operating time | 19 | 2112 | Random | | 9.94 | | 6.07,13.81 | | <0.00001 | 81 |
| Pre 2000 | 12 | 1346 | Random | | 11.56 | | 6.05,17.06 | | <0.0001 | 82 |
| Post 2000 | 7 | 766 | Random | | 6.09 | | 2.14,10.04 | | 0.003 | 59 |
| Postoperative hospital stay | 19 | 2761 | Random | | -0.50 | | -0.82,-0.19 | | 0.002 | 95 |
| Pre 2000 | 11 | 1843 | Random | | -0.55 | | -0.86,-0.24 | | 0.0005 | 85 |
| Post 2000 | 8 | 918 | Random | | -0.30 | | -0.81,-0.21 | | 0.25 | 82 |
| Return to normal activity | 13 | 1613 | Random | | -3.19 | | -4.64,-1.75 | | <0.0001 | 89 |
| Pre 2000 | 8 | 1198 | Random | | -4.72 | | -5.99,-3.46 | | <0.00001 | 77 |
| Post 2000 | 5 | 415 | Random | | -0.56 | | -2.09,0.97 | | 0.47 | 54 |
| Resumption of normal diet | 8 | 1165 | Random | | -0.43 | | -0.63,-0.24 | | <0.0001 | 95 |
| Pre 2000 | 4 | 621 | Random | | -0.22 | | -0.39,-0.06 | | 0.009 | 79 |
| Post 2000 | 4 | 544 | Random | | -0.53 | | -0.68,-0.38 | | <0.00001 | 47 |
| VAS for postoperative pain | 5 | 892 | Random | | -0.54 | | -0.71,-0.37 | | <0.00001 | 35 |
| Pre 2000 | 3 | 740 | Random | | -0.45 | | -0.80,-0.10 | | 0.01 | 67 |
| Post 2000 | 2 | 152 | Random | | -0.55 | | -0.78,-0.32 | | <0.00001 | 0 |

**Table S4: Meta-analysis of the complications in all studies**

| Outcome | Studies reporting outcome | | Meta-analysis | | | | |
| --- | --- | --- | --- | --- | --- | --- | --- |
|  | Studies | Patients | Type | Odds Ratio | 95% CI | *P* | I^2^ (%) |
| **Wound infection** | 31 | 4063 | Fixed | 0.45 | 0.34,0.59 | <0.00001 | 0 |
| **Pre 2000** | 18 | 1993 | Fixed | 0.36 | 0.23,0.54 | <0.00001 | 0 |
| **Post 2000** | 13 | 2070 | Fixed | 0.53 | 0.37,0.76 | 0.0005 | 13 |
| **Intra-abdominal abscess** | 17 | 3445 | Fixed | 1.56 | 1.01,2.43 | 0.05 | 2 |
| **Pre 2000** | 8 | 1612 | Fixed | 1.59 | 0.75,3.38 | 0.22 | 0 |
| **Post 2000** | 9 | 1833 | Fixed | 1.55 | 0.90,2.68 | 0.12 | 42 |
| **Postoperative ileus** | 18 | 3206 | Fixed | 0.91 | 0.57,1.47 | 0.71 | 0 |
| **Pre 2000** | 12 | 1715 | Fixed | 1.28 | 0.71,2.31 | 0.42 | 0 |
| **Post 2000** | 6 | 1491 | Fixed | 0.47 | 0.20,1.12 | 0.09 | 16 |
| **Intraoperative bleeding** | 4 | 779 | Fixed | 1.56 | 0.54,4.48 | 0.41 | 0 |
| **Urinary tract infection** | 5 | 796 | Fixed | 1.76 | 0.58,5.29 | 0.32 | 0 |
